# Eccrine Spiradenoma of the Lower Back: A Surgical Case Report

**DOI:** 10.7759/cureus.70499

**Published:** 2024-09-30

**Authors:** Ayman Nadeem, Mukarram Mohammed Abdul, Madhu Chandana Reddy Pulakanti, Aditi Agarwal, Kashif Uddin Ahmed

**Affiliations:** 1 General Surgery, Osmania Medical College, Hyderabad, IND

**Keywords:** adnexal tumor, benign skin tumor, differential diagnosis, eccrine sweat gland tumor, histopathology, sebaceous cyst, spiradenoma, subcutaneous nodule, tumor pathology, wide local excision

## Abstract

Spiradenoma is an uncommon benign neoplasm originating from eccrine sweat glands, typically characterized by a solitary tumor. Its diagnosis poses a challenge due to its morphological similarity to other solitary swellings. This report highlights the case of a 28-year-old male who presented with a solitary swelling on his right lower back. On ultrasonography, it was initially diagnosed as a sebaceous cyst. However, surgical excision and histopathological analysis revealed a well-defined grey-white nodule composed of basaloid cells organized in an alveolar and tubular pattern. This confirmed the diagnosis of eccrine spiradenoma, underscoring the critical importance of accurate diagnosis and management.

The report emphasizes the significance of histopathology in differentiating eccrine spiradenoma from other solitary swellings and identifying its malignant potential. Furthermore, regular follow-up is necessary to prevent recurrence and potential metastasis. There is a need for further research to enhance the understanding and management of spiradenoma, along with its long-term outcomes.

## Introduction

Spiradenomas are benign adnexal tumors of eccrine sweat glands that arise from the differentiation of their ductal and secretory components [1]. These tumors are uncommon, and the precise incidence of benign solitary spiradenomas remains unknown. Malignant spiradenomas are rare, with approximately 120 documented cases worldwide [2]. In our case, a solitary nodule on the lower back was initially diagnosed as a sebaceous cyst but was later identified as a spiradenoma. Therefore, it is important to be aware of the different clinical, imaging, and histopathological findings associated with spiradenomas for improved diagnosis and management.

## Case presentation

A 28-year-old male presented with swelling in the right lower back for two years, which had an insidious onset and gradually progressed to its current size over three months. The swelling was associated with sharp, stinging pain when touched or manipulated. There was no history of fever, and both past and family histories were insignificant.

Local examination of the lower back revealed a swelling measuring 1.5×1.5 cm with well-defined margins, located approximately 3 cm to the right of the lumbar spine. The swelling was hard in consistency and freely mobile at its base.

The skin over the swelling was mildly pigmented but not pinchable. There were no visible pulsations, discharge, or local rise in temperature. An ultrasonography scan showed a hypoechoic lesion in the subcutaneous plane without any vascularity, initially diagnosed as a sebaceous cyst. To confirm the diagnosis, fine needle aspiration was performed on the swelling, and the sample was sent to the laboratory for a cytosmear study. This revealed small clusters of epithelial cells with round to oval nuclei and scant to moderate amounts of cytoplasm. Numerous bare nuclei and a few spindle cells were present in a hemorrhagic background, suggesting an adnexal lesion. Therefore, excision was advised for a definitive diagnosis of the mass. 

Under local anesthesia, a 3 cm skin incision was given, extended subcutaneously to reach the swelling, and delineated all over to create a plane. The mass was then exposed and all adhesions were removed. Holding it with Babcock's forceps, pedicle adhesions were dissected and excised. It had a relatively harder consistency on closer inspection. Subsequently, it was sent for histopathology assessment, and the skin was closed using 3-0 non-absorbable sutures.

**Figure 1:**
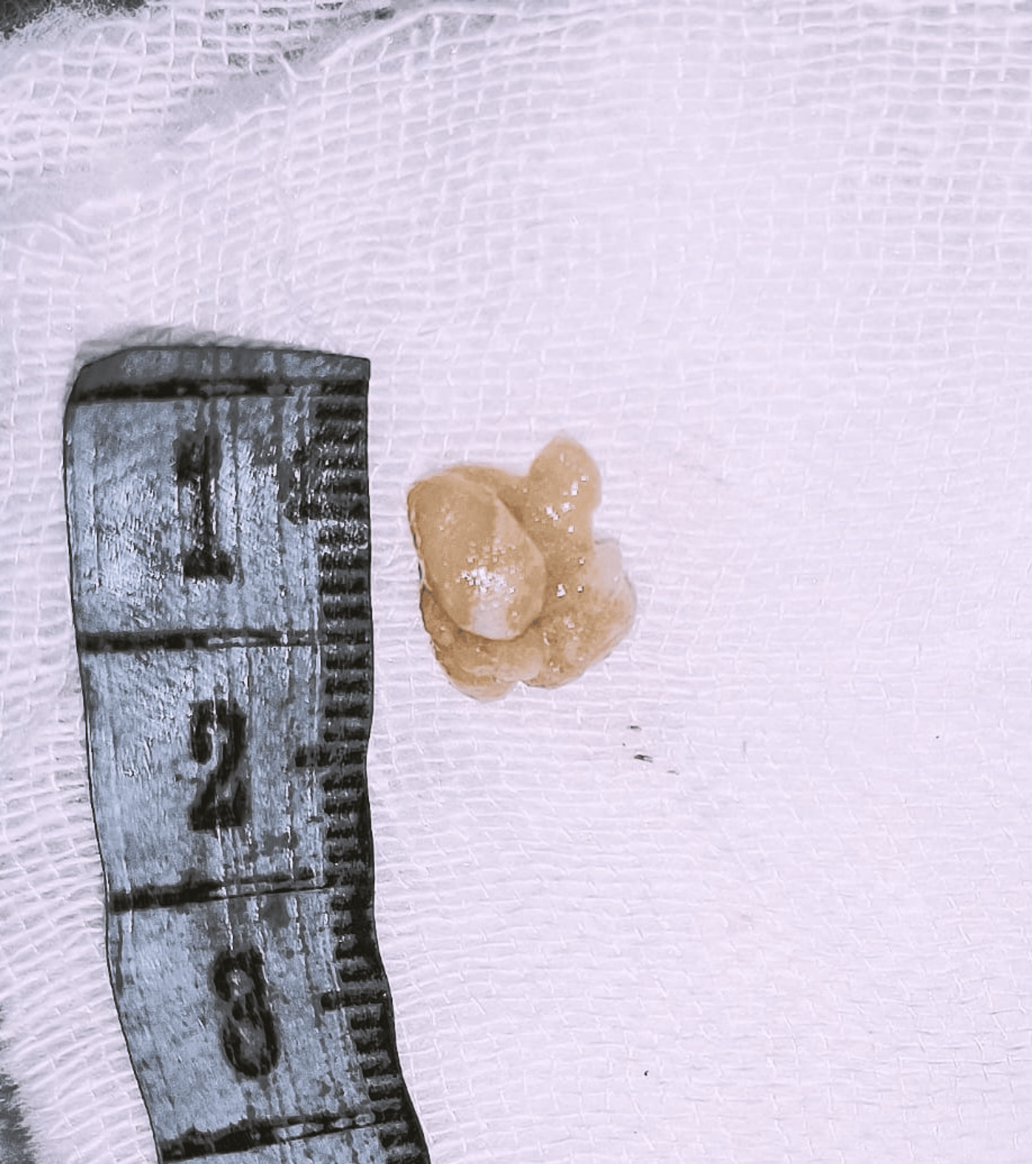
Intraoperative gross picture of the excised specimen, measuring 1×0.5 cm

As shown in Figure 1, grossly, it appeared as soft tissue bits, one being 1×0.5 cm and the other being 1.5×0.5 cm. The external surface showed nodularity. The cut section showed a well-circumscribed grey-white nodule covered by a thin fibrous capsule.

**Figure 2:**
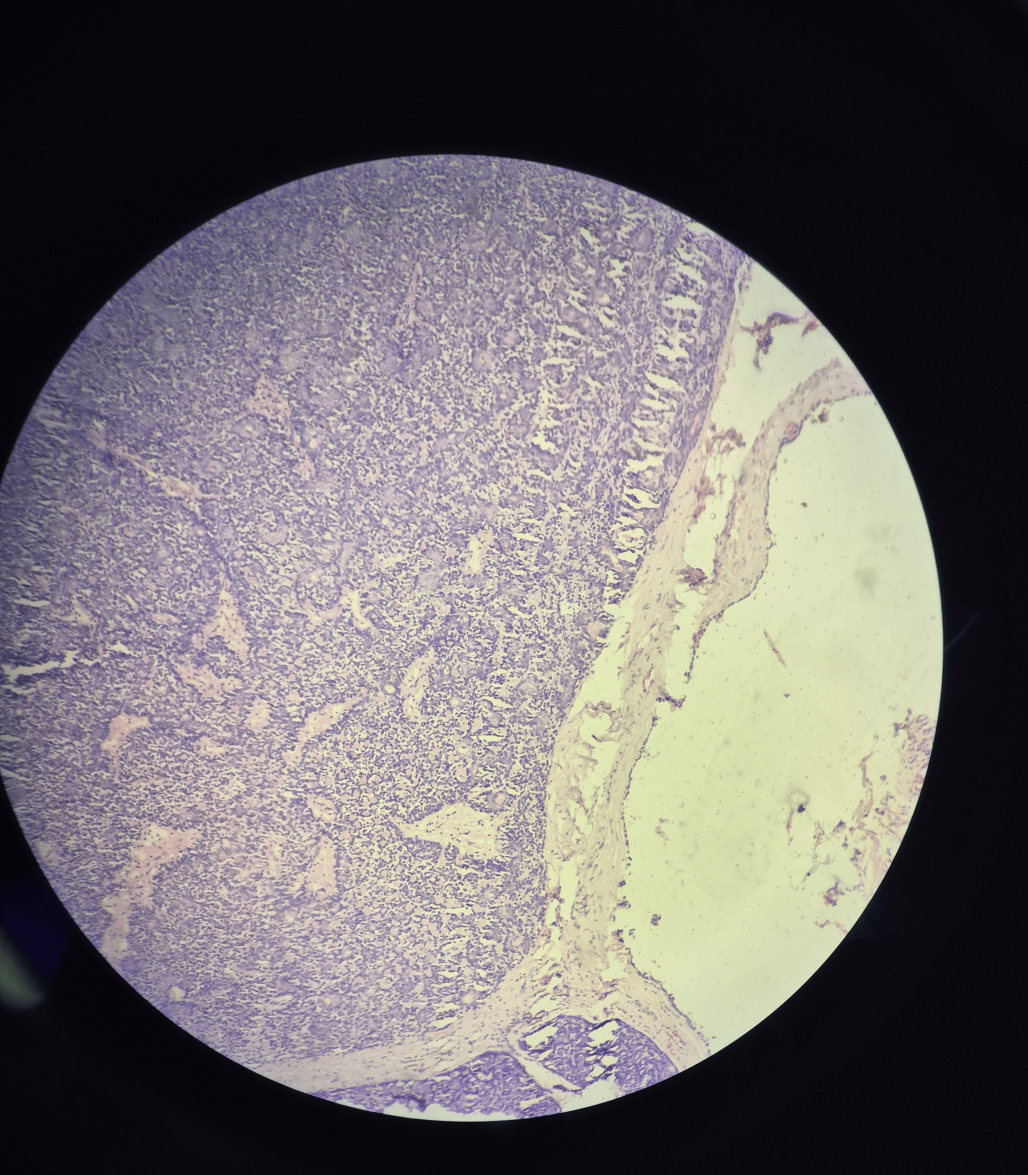
Histology of the tumor, showing a well-circumcised nodule covered by a thin fibrous capsule consisting of basaloid cells arranged in an alveolar and tubular pattern, with the absence of mitoses and atypia

Histopathological examination, as shown in Figure 2, revealed basaloid cells arranged in an alveolar and tubular pattern. Two distinct sizes of cells were noted; larger cells had clear cytoplasm with vesicular nucleus and smaller cells were dark and basaloid. The tubules were lined by tall columnar to cuboidal epithelium with basement membrane. Mitoses and atypia were absent, further confirming the diagnosis.

## Discussion

A benign tumor of the skin adnexa arising from the eccrine sweat gland is called spiradenoma, first described by Kersting and Helwig [3]. It is most commonly seen in individuals aged 15-35 years and usually presents as a single solitary painful nodule ranging in size from 0.3 cm to 5 cm [1], typically located on the face or upper trunk. When presented as multiple nodules, the patterns observed can be linear, zosteriform, nevoid, or Blaschkoid [4]. In this case, the lesion was found on the right lower back, approximately 3 cm to the right of the lumbar spine, making it a relatively unusual location.

Ultrasonography revealed a hypoechoic lesion in the subcutaneous plane that closely mimics a sebaceous cyst. However, ultrasonography alone cannot provide a definitive diagnosis. A cytosmear study, supplemented with histopathology, was necessary for accurate diagnosis.

The histopathologic findings in this case indicated a benign adnexal lesion with well-defined borders and the absence of atypia and mitoses, which is crucial for assessing the malignant potential of spiradenoma. It is also important to distinguish eccrine spiradenoma from other masses such as glomus tumors, angioleiomyoma, and cylindromas due to significant histopathological similarities [1,5]. While treatment for spiradenoma is not clearly established, wide local excision is considered the gold standard. Early and accurate diagnosis is crucial to prevent recurrence and identify potential malignant transformation. Regular follow-up is essential to detect recurrence early, allowing for timely surgical resection and preventing metastases [6].

## Conclusions

In summary, we report a relatively rare case of a benign adnexal tumor of eccrine sweat glands, which typically presents as a solitary painful nodule and is most commonly found on the face or upper trunk. The location on the lower back in this case is unusual. Histopathology plays a crucial role in confirming the diagnosis and distinguishing it from similar conditions. Treatment generally involves wide local excision, and regular follow-up is important to monitor for potential recurrence. Further research and studies are needed to better understand the management and long-term outcomes of this rare condition.

## References

[1] Xing Y, Wu X, Xu C (2021). Ultrasonographic features of eccrine spiradenoma: a case report. Medicine (Baltimore).

[2] Tazi Z, Ngalande E, Fdili FZ, Jayi S, Chaara H, Melhouf MY (2024). Eccrine spiradenoma. World Journal of Advanced Research and Reviews.

[3] Kersting DW, Helwig EB (1956). Eccrine spiradenoma. AMA Arch Derm.

[4] Dhua S, Sekhar DR (2016). A rare case of eccrine spiradenoma-treatment and management. Eur J Plast Surg.

[5] Sharma A, Sengupta P, Das AK, Nigam MK, Chattopadhya S (2014). Eccrine spiradenoma in knee. Indian J Dermatol.

[6] Wagner K, Jassal K, Lee JC, Ban EJ, Cameron R, Serpell J (2021). Challenges in diagnosis and management of a spiradenocarcinoma: a comprehensive literature review. ANZ J Surg.

